# Peripheral Neuropathy as a Complication of Diabetic Ketoacidosis in a Child with Newly Diagnosed Diabetes Type 1: A Case Report

**DOI:** 10.4274/jcrpe.5374

**Published:** 2018-07-31

**Authors:** Marta Baszyńska-Wilk, Marta Wysocka-Mincewicz, Anna Świercz, Jolanta Świderska, Magdalena Marszał, Mieczysław Szalecki

**Affiliations:** 1 The Children’s Memorial Health Institute, Clinic of Endocrinology and Diabetology, Warsaw, Poland; 2 Jan Kochanowski University, The Faculty of Medicine and Health Sciences, Kielce, Poland

**Keywords:** Polyneuropathy, ketoacidosis, diabetes mellitus type 1, children

## Abstract

Neurological complications of diabetic ketoacidosis are considered to be a serious clinical problem. The most common complication is cerebral edema. However, these neurological complications also include less common entities such as ischemic or hemorrhagic stroke, cerebral venous and sinus thrombosis or peripheral neuropathy.

We present a case of a 9-year old girl admitted to our intensive care unit with new onset type 1 diabetes, diabetic ketoacidosis, cerebral edema, multifocal vasogenic brain lesions and bilateral lower limb peripheral paresis. The patient developed polydipsia and polyuria one week before admission. The initial blood glucose level was 1136 mg/dL and severe acidosis was present (pH 7.1; BE-25.9). Computed tomography scan showed brain edema and a hypodense lesion in the left temporal region. Brain magnetic resonance imaging revealed more advanced multifocal brain lesions. Nerve conduction studies demonstrated damage of the motor neurons in both lower limbs with dysfunction in both peroneal nerves and the right tibial nerve. With treatment and physiotherapy, the patient’s health gradually improved.

Acute neuropathy after ketoacidosis is a rare complication and its pathogenesis is not clear. Patients with diabetic ketoacidosis require careful monitoring of neurological function, even after normalization of their glycemic parameters.

## What is already known on this topic?

Neurological complications of ketoacidosis in diabetes mellitus type 1 are serious clinical problems. Neuropathy after ketoacidosis in children is extremely rare. Only a small number of cases with this complication have been reported.

## What this study adds?

This paper presents the current state of knowledge about peripheral neuropathy in pediatric patients with new-onset type 1 diabetes and includes clinical presentation, pathophysiology and available treatment for this rare complication.

## Introduction

Neurological complications of ketoacidosis in diabetes mellitus type 1 (DM1) present a serious clinical problem. Brain edema is the most common central nervous system (CNS) complication which occurs in approximately 0.5-1% of cases with diabetic ketoacidosis (DKA) and has a 20% mortality rate (1,2). Ischemic and hemorrhagic strokes are less common and account for 10% of intracerebral complications of DKA (3). Cerebral vein and sinus thrombosis are also less frequent than brain edema while neuropathy after DKA is extremely rare.

## Case Report

We present a 9-year-old girl with newly diagnosed DM1, DKA, brain edema, multifocal vasogenic brain lesions and lower limb paresis.

The patient was reported to have polyuria and polydipsia over the past week and a weight loss of 3 kg over the previous month. The patient was admitted to the district hospital in a serious clinical condition with severe dehydration. Initial intravenous fluid therapy included infusion of 15 mL/kg of 0.9% sodium chloride during the first 90 minutes. The total volume of fluids administered during the first 12 hours and consisting of 1250 mL 0.9% sodium chloride (NaCI) and 1000 mL of 5% dextrose with 0.9% NaCI (2:1 proportion, sodium concentration-51.34 mEq/L) was 65 mL/kg (patient’s weight-34.6 kg). Intravenous insulin therapy was introduced in an initial dose of 0.05 units/kg/hour in order to prevent a rapid decrease of glycaemia. After three hours, the patient’s medical state and neurological condition was reported to deteriorate. She experienced motor restlessness and agitation followed by upper limb spasms. At the end of the first day of treatment the patient was transferred to the Intensive Care Unit (ICU) of the Children’s Memorial Health Institute in Warsaw with a Glasgow Coma Scale (GCS) score of 13 points. Results of laboratory tests are shown in Table 1.

**Figure 1:**
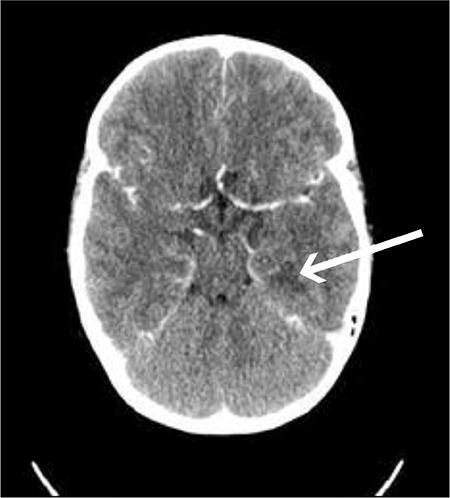
Brain computed tomography scan. Thirteen mm hypodense lesion in the left temporal lobe, not visible after contrast injection-ischemic lesion? The supratentorial ventricular system is narrow and symmetrical. Cerebral sulci are not distinct

Six hours after admission to the ICU, her clinical state was deteriorating rapidly and the GCS score had decreased to 7 points. Computed tomography scan revealed brain edema and a 13-mm hypodense lesion in the left temporal region (Figure 1). 

**Figure 2:**
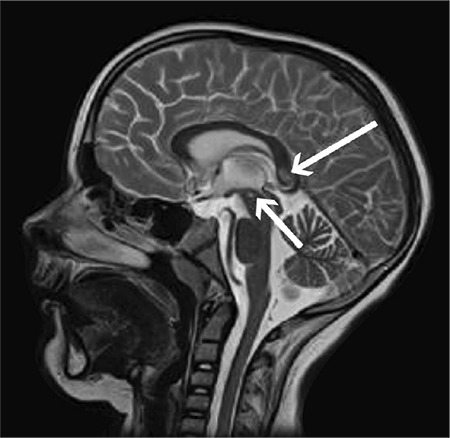
Brain magnetic resonance imaging. T1-weighted scan. Lesions located in the corpus callosum and the midbrain

**Figure 3:**
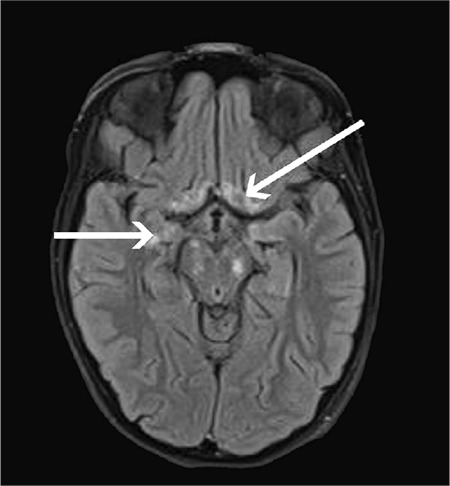
Brain magnetic resonance imaging. Fluid-attenuated inversion recovery sequence. Lesions located in the medial parts of the temporal lobes and in the lower-medial area of the frontal lobes and the ventricles

The patient was sedated and intubated. Insulin infusion was continued and intravenous fluid administration was diminished. Anti-edematous treatment was introduced (Mannitol 0.3 g/kg/dose, three times per day). The patient’s state showed a gradual improvement. After four days, she was extubated. Subsequently the patient was transferred to the Department of Endocrinology and Diabetology. Despite improvement in her clinical condition, the patient was found to have developed symmetric lower limbs paresis. Brain magnetic resonance imaging (MRI) revealed numerous, diffuse lesions (Figure 2, 3). Presence of infection and neoplasm of CNS were ruled out.

Lower limb nerve conduction studies (NCS) revealed damage to the motor neuron in both lower extremities with dysfunction in both peroneal nerves and in the right tibial nerve. Neurological opinion was that the etiology of the multifocal brain lesions was vasogenic. However, the cause of neuropathy was not fully clear. Presence of DKA and peripheral ischemia were given as the probable factors leading to development of the neuropathy.

Alpha lipoic acid and vitamins B1, B6 and B12 were introduced to the therapeutic regimen and the patient underwent intensive physiotherapy, which led to improvement of left lower limb motor function.

Brain MRI was performed three months later in which no progression in size and number of the brain lesions was observed. NCS revealed normalization of the left peroneal nerve parameters. However, findings indicative of deep motor neuropathy of the right lower limb was found to persist. An informed consent form for publication was given by the parents.

## Discussion

Diabetic neuropathy (DN) refers to the presence of symptoms and/or signs of peripheral nerve dysfunction due to diabetes. In order to make a diagnosis of DN, other neuropathic etiologies should be excluded which include vitamin deficiency, infection, inflammatory causes, toxic, autoimmune, paraneoplastic and genetic causes (4). Neuropathy is the most common complication of diabetes and is encountered in approximately 45% patients with DM2 and 54-59% patients with DM1 (5). Due to different symptoms, clinical courses and pathogenic mechanisms, DN is considered a heterogeneous entity which includes many types of nerve dysfunction. More than 80% of patients with symptomatic DN suffer from generalized symmetric, chronic polyneuropathy including motor, sensory and/or autonomic nerve dysfunctions. In most cases DN develops over the years in patients with long standing hyperglycemia (6). However, there are also few types of neuropathy related to newly diagnosed DM1, which are extremely rare and there is scarce data on this issue (7). They can be classified as acute painful DN, hyperglycemic neuropathy and neuropathy after ketoacidosis. It is interesting that not only symptomatic but also asymptomatic changes in nerve function can be seen at the time of DM1 diagnosis. Lee et al (8) examined nerve conduction in children with newly diagnosed DM1 and periodically during their 5-year follow-up. This prospective study included patients aged 3-19 years (n=37), who underwent bilateral NCS of median, ulnar, posterior tibial, peroneal and sural nerves annually for five years. In 32.4% of the patients the examination revealed electrophysiological evidence of polyneuropathy in at least two different nerves at the time of diagnosis of DM1. Dayal et al (9) presented a case report of a 12-year-old girl who developed acute, asymmetric, sensorimotor neuropathy during the first month after diagnosis of DM1. During this time, the authors observed reduction of hemoglobin A1c (HbA1c) from 14.2% to 10.4% which was described as a potential trigger factor for neuropathy. Wilson et al (10) presented a similar case of DN. A 14-year-old boy was diagnosed with DM1. After nine weeks of diabetes treatment the authors observed HbA1c level reduction from 14.1% to 7.6%. These two cases present clinical features of acute painful DN, also known as a treatment-induced neuropathy (insulin neuritis). This is a reversible disorder and affects diabetes patients who show a rapid improvement of metabolic control and have a favorable clinical outcome (11). In our patient, this diagnosis was taken into consideration however the decrease of the HbA1c level was not that significant as in two cases above (HbA1c level at diagnosis was 11.5% and one month later it was 9.6%).

Another type of DN is hyperglycemic neuropathy. Its clinical presentation includes temporary limb hyperalgesia which is potentially reversible after glycemic normalization (12).

Rangel et al (7) described a 10-year-old patient with new onset DM1 and concomitant acute mononeuropathy which manifested in difficulty in flexing the right foot and hyperalgesia, extending from the dorsum of the right foot to the ankle. According to these authors, DM was responsible for this mononeuropathy as its onset was simultaneous with the onset of DM1. Motor dysfunction rapidly improved after adequate glycemic control. 

Our patient suffered from acute motor peripheral neuropathy, which was probably caused by DKA. This neuropathy can be a consequence of the peripheral ischemia or hemodynamic and metabolic changes linked to the ketoacidosis (13). One hypothesis is that the procoagulant state which occurs during DKA can cause nerve damage through vascular endothelial dysfunction, which is the first line of defense against thrombosis. Endothelial damage also leads to platelet and coagulation factor activation (14,15). The plasma levels of fibrinogen, factors VII, VIII, XI, XII and von Willebrand are elevated in DKA. A procoagulant state is worsened by disrupted anticoagulant mechanisms, such as a low protein C level. Fibrinolysis is also impaired due to different factors such as more difficult degradation of the thrombi or an increased concentration of plasminogen activator inhibitor type 1 (14,15). The patient in our report had slightly elevated d-dimer concentrations. However, further diagnostic tests were not conducted. In our case other etiologies of neuropathy, such as hypophosphatemia, which was observed during admission to the district hospital, were also taken into consideration. Hypophosphatemia is generally asymptomatic. Nonetheless, its severe form (<0.32 mmol/L) can lead to peripheral polyneuropathy which may be both motor and sensory (16). In our patient, neurological symptoms were present even after normalization of the phosphate level suggesting an alternative mechanism. Differential diagnosis also included Guillain-Barré syndrome, but no typical pathology in cerebrospinal fluid was seen.

The treatment of DN includes use of strong antioxidants such as alpha lipoic acid. The effectiveness of this therapy was proven in meta-analyses (17,18). Benfotiamine, a derivative of vitamin B1, was also shown to increase the utilization of active glycolysis products, although less effectively than alpha lipoic acid (19).

In conclusion, the findings in this patient indicate that neuropathy is not only seen as a late complication of diabetes mellitus, but that it can develop any time after, or even before DM1 diagnosis (6). However, acute neuropathy after ketoacidosis is a rare complication and its pathogenesis is not clear. Patients with DKA require careful monitoring of neurological functions even after normalization of glycemic parameters (20).

## Figures and Tables

**Table 1:**
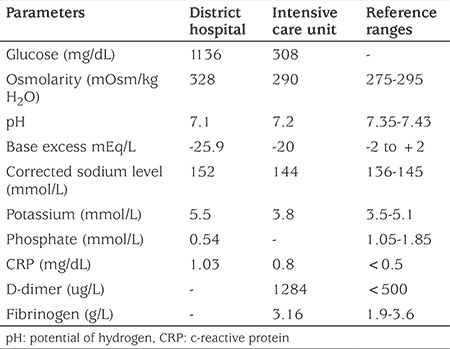
Results of laboratory tests during admission to the district hospital and to the Intensive Care Unit of the Children’s Memorial Health Institute
